# Clinical and Esthetic Outcomes of Vestibular Socket Therapy: A Systematic Review and Meta-Analysis

**DOI:** 10.3390/dj14090538

**Published:** 2026-08-29

**Authors:** Yasser Sherif Soliman, Nadine Gräfin von Krockow, Fatma E. A. Hassanein, Mihad Ibrahim, Abdelsalam ElAskary, Hossam Ismail

**Affiliations:** 1 Oral and Maxillofacial Prosthodontics Department, Goethe University, 60323 Frankfurt, Germany; yasser_sherif@dent.asu.edu.eg; 2 Oral and Maxillofacial Prosthodontics Department, Ain Shams University, Cairo 11566, Egypt; 3 Department of Postgraduate Education, Goethe University, 60323 Frankfurt, Germany; krockow@med.uni-frankfurt.de; 4 Oral Medicine, Periodontology and Oral Diagnosis, Faculty of Dentistry, King Salman International University, El-Tur 46612, Egypt; fatma.hassanein@ksiu.edu.eg; 5 Department of Periodontology, Faculty of Dentistry, Cairo University, Cairo 11553, Egypt; 6 ElAskary and Associates, Alexandria 21500, Egypt; askary@askaryimplants.com; 7 School of Dentistry, New York University, New York, NY 10012, USA; 8 School of Dentistry, University of Western Australia, Perth, WA 6009, Australia; hossam.ismail@uwa.edu.au

**Keywords:** vestibular socket therapy, immediate implant placement, pink esthetic score, marginal bone level, esthetic outcomes

## Abstract

**Background/Objectives:** Vestibular socket therapy (VST) is a minimally invasive technique for immediate implant placement (IIP) in compromised extraction sockets that reconstructs the deficient buccal plate while preserving the crestal soft tissue. Despite growing clinical adoption, the randomized evidence on its predictability and biological performance remains limited. **Methods:** Following PRISMA 2020 and prospectively registered in PROSPERO (CRD420261288724), PubMed/MEDLINE and EMBASE were searched to January 2026 for randomized controlled trials (RCTs) evaluating VST with IIP. Risk of bias (RoB 2) and certainty of evidence (GRADE) were assessed. Final follow-up buccal bone plate thickness was pooled using a random-effects model; trials comparing variations within VST were reported narratively but not pooled. **Results:** Seven RCTs (186 participants) were included; implant survival was 99.5%, with no shield exposure, peri-implantitis, or dehiscence. Meta-analysis of the three trials comparing VST against a distinct alternative technique showed no significant between-group difference in final buccal bone plate thickness at any level: crestal +0.24 mm (95% CI−0.13 to 0.61; I^2^ = 45%), mid-root +0.32 mm (−0.45 to 1.09; I^2^ = 82%), or apical +0.19 mm (−0.48 to 0.87; I^2^ = 67%). VST showed minimal mid-facial recession, stable papillae, and Pink Esthetic Scores of 10.29–13.17. Certainty was very low for all outcomes. **Conclusions:** VST achieved high implant survival and favorable soft tissue and esthetic outcomes but did not significantly outperform comparator techniques in buccal bone plate thickness, with very low certainty. Adequately powered, independent trials with longer follow-up are needed.

## 1. Introduction

Dental implants are a predictable and widely accepted treatment modality for replacing missing teeth, providing long-term functional stability and favorable esthetic outcomes Sartoretto et al., 2023 [1]. Immediate implant placement (IIP), defined as implant insertion into a fresh extraction socket, has emerged as an attractive alternative to early or delayed placement protocols, offering advantages such as reduced treatment time, fewer surgical interventions, and improved patient satisfaction Seyssens et al., 2022 [2]. However, the success of IIP remains biologically and surgically demanding Yankov, 2023 [3], particularly in the maxillary esthetic zone, where post-extraction remodeling of the alveolar ridge is inevitable Tan et al., 2012; Chappuis et al., 2017 [4,5]. The inevitable resorption of the thin buccal bone plate may result in mid-facial gingival recession, loss of interdental papillae, and compromised long-term esthetic outcomes Chappuis et al. 2017, 2015 [5,6].

Post-extraction alveolar remodeling leads to substantial dimensional changes of the ridge, with horizontal bone loss reported to average 3.5–5 mm and vertical reduction of approximately 1.5–2 mm during the first six months following extraction Couso-Queiruga et al. 2021 [7]. Moreover, IIP alone does not prevent this physiologic remodeling process Clementini et al. 2015 [8]. Consequently, numerous surgical strategies have been proposed to mitigate buccal bone resorption and preserve peri-implant tissue architecture during IIP Bakkali et al. 2021 [9]. These include particulate grafting of the jumping gap Grunder 2011 [10], guided bone regeneration (GBR) Retzepi & Donos 2010 [11], socket shield or partial extraction therapy (PET) Hürzeler et al. 2010 [12], and the dual-zone (DZ) concept Chu et al. 2012 [13].

Vestibular Socket Therapy (VST) has been proposed as a minimally invasive technique to treat compromised sockets using IIP Elaskary et al. 2020 [14] without compromising the crestal soft tissue integrity via a vestibular access incision while simultaneously reconstructing the deficient buccal plate using a flexible cortical xenogeneic membrane designed to stabilize the grafting material Elaskary et al. 2021 [15]. Post-extraction sockets are commonly categorized by the integrity of the residual facial bone and overlying soft tissue: Type I sockets retain an intact facial bone wall beneath healthy soft tissue, whereas Type II sockets are characterized by partial or complete loss of the facial bone plate beneath intact overlying soft tissue Elian et al., 2007 [16].

The biological rationale underlying VST centers on the hypothesis that vestibular tunnel access, combined with a cortical membrane barrier, may reduce periosteal disruption at the crestal margin while simultaneously providing structural support for the grafted buccal plate. This approach has been proposed to offer shorter treatment timelines, reduced mid-facial recession, and improved esthetic stability compared with conventional flap-based immediate implant protocols Elaskary et al., 2021 [15]; however, these proposed advantages require validation through independent clinical evidence.

Early clinical evidence from a limited number of trials suggests that VST may be associated with favorable hard and soft tissue outcomes and improved esthetic stability Hamed et al., 2023; Elaskary et al., 2024 [17,18]. However, this evidence is recent, heterogeneous, and of limited methodological quality, so biological plausibility cannot yet be equated with established clinical effectiveness. Although systematic reviews have examined immediate implant placement in compromised extraction sockets more broadly Campi et al., 2025 [19], none have focused specifically on VST or synthesized its randomized evidence. Therefore, this systematic review and meta-analysis aimed to evaluate the clinical effectiveness of VST in conjunction with IIP in terms of buccal bone plate thickness, implant survival, and soft tissue stability.

## 2. Materials and Methods

### 2.1. Protocol and Registration

Before undertaking this systematic review and meta-analysis, a detailed protocol was developed in accordance with the PRISMA 2020 checklist Page et al., 2021 [20] (Table S2). This systematic review was designed and reported in accordance with the Preferred Reporting Items for Systematic Reviews and Meta-Analyses (PRISMA) 2020 statement. The review protocol was prospectively registered in the International Prospective Register of Systematic Reviews (PROSPERO) Registration No. CRD420261288724 on 19 January 2026. The review process commenced on 19 January 2026, before formal data extraction and analysis.

### 2.2. Study Question and PICO Framework

The present systematic review was designed to address the following research question, formulated according to the PICO framework:

In adults with compromised fresh extraction sockets, does IIP using VST improve hard and soft tissue outcomes and esthetic performance compared with conventional immediate or early implant placement techniques?

**Population:** Patients aged >18 years, presenting with compromised fresh extraction sockets, including infected and non-infected sites.
**Intervention:** IIP performed using VST, with or without adjunctive grafting procedures.
**Comparison:** Clinically relevant comparators including conventional IIP techniques, early implant placement with contour augmentation, dual-zone grafting, and partial extraction therapy.
**Outcomes:** Final buccal bone plate thickness, soft tissue stability, gingival recession, implant survival, and esthetic performance, assessed using the Pink Esthetic Score (PES).

### 2.3. Eligibility Criteria

Eligible studies were those published between 2020 (when VST was first described) and the last search date (January 2026) that reported outcomes of VST performed in conjunction with IIP.

Included study designs comprised randomized controlled trials (RCTs) whose participants were required to be adults presenting with non-restorable single teeth in the esthetic zone and exhibiting thin or deficient labial bone walls covered by soft tissue. Studies were included if they assessed hard tissue outcomes, soft tissue outcomes, implant survival, esthetic parameters, or combinations thereof.

With respect to comparator groups, no restriction was placed on the nature of the control intervention, provided it represented a recognized clinical approach to socket management at the time of IIP. This inclusive comparator strategy was adopted because the primary aim was to evaluate VST’s clinical performance across the range of techniques it may realistically replace or complement in practice rather than against a single biologically homogeneous control. Importantly, comparators were not assumed to be biologically equivalent. In particular, PET was included as a clinically relevant active comparator in the specific context of intact thin-walled Type I sockets; its biological mechanism—preservation of the buccal root fragment, bundle bone, periodontal ligament fibers, and local vasculature—is fundamentally distinct from VST’s de novo buccal plate reconstruction approach. Also, the early implant placement and contour augmentation were used as a comparator despite being type 2 as opposed to IIP, which is type 1, as it is a well-established procedure in managing defective sockets. This distinction was preserved throughout data extraction, narrative synthesis, and meta-analytic subgrouping and is explicitly reflected in the interpretation of pooled and study-level findings.

Studies were excluded if they were: non-randomized prospective studies, case series, case reports, retrospective analyses, animal studies, or in vitro studies, or if VST was not clearly defined according to the original protocol by Elaskary et al. 2020 [14]. Reviews, editorials, and conference abstracts without full-text data were also excluded.

### 2.4. Outcome Measures

The primary outcome measure was the final buccal bone plate thickness, defined as the horizontal (bucco-oral) dimension of the facial osseous plate measured on cone-beam computed tomography (CBCT) cross-sectional slices oriented perpendicular to the long axis of the implant at three apico-coronal levels (crestal, mid-implant, and apical), from baseline to final follow-up (6–12 months). For trials whose primary endpoint was reported under a bucco-palatal ridge designation, only the facial bone plate component (not total ridge width) was extracted and pooled so that all pooled values represent the same anatomical structure.

Secondary outcomes included implant survival at 12–24 months, soft tissue stability, mid-facial recession and papilla position, peri-implant soft tissue contour, and esthetic outcomes evaluated using the PES. Patient-reported satisfaction and postoperative experience were recorded when available.

### 2.5. Search Methods for the Identification of Studies

A systematic electronic search was conducted in PubMed/MEDLINE and EMBASE from database inception to the search date, January 2026. The search strategy was developed and combined MeSH terms and free-text keywords. The complete search strategies for PubMed/MEDLINE and EMBASE, including all search terms, Boolean operators, field tags, filters, and date parameters, are provided in full in Supplementary File S1 and Supplementary File S2. No language restrictions were applied to the electronic search.

Reference lists of all included studies and relevant systematic reviews were hand-searched. Grey literature was searched via ClinicalTrials.gov and the WHO International Clinical Trials Registry Platform (ICTRP). No restrictions were applied for language or year of publication. PubMed/MEDLINE and EMBASE were selected, as they jointly index the majority of the peer-reviewed biomedical and dental literature.

### 2.6. Study Selection

Two independent reviewers (M.I. and Y.S.) screened titles and abstracts against the pre-defined eligibility criteria. Full texts of potentially eligible studies were retrieved and assessed independently by the same reviewers. Disagreements were resolved by consensus and, if necessary, arbitration by a third reviewer. A PRISMA 2020 flow diagram documents the selection process.

### 2.7. Data Extraction

Data were extracted by two reviewers independently using a pre-piloted standardized extraction form. The following data items were collected: study identification (first author, year, journal), study design and registration, setting, sample size, participant characteristics (age, sex, socket classification, tooth type), comparator groups, implant specifications, grafting materials, membrane type, follow-up duration, and all reported outcome data (means, standard deviations, medians, interquartile ranges, *p*-values). Authors were contacted when outcome data were missing or unclear.

To address the potential for participant overlap across trials originating from the same investigator network, recruitment periods, clinical settings, and sample sizes were cross-examined for all included studies during data extraction. Where recruitment timelines were not explicitly reported in the published manuscripts, the corresponding authors were contacted directly for clarification. Only trials that represented independent patient cohorts with no shared participants across studies were included.

### 2.8. Risk of Bias and Quality Assessment

Risk of bias assessment was performed independently by two reviewers (M.I. and Y.S.). Randomized controlled trials were evaluated using the Cochrane Risk of Bias 2 (RoB 2) tool Flemyng et al., 2020 [21], assessing bias arising from the randomization process, bias due to deviations from intended interventions, bias due to missing outcome data, bias in measurement of the outcome, and bias in selection of the reported result. The certainty of the evidence for each outcome was assessed using the Grading of Recommendations, Assessment, Development and Evaluation (GRADE) approach Goldet & Howick 2013 [22]. Evidence profiles were generated using the GRADEpro Guideline Development Tool (GDT). Each outcome was rated across five domains: risk of bias, inconsistency, indirectness, imprecision, and other considerations, including publication bias, and assigned an overall certainty rating of high, moderate, low, or very low.

### 2.9. Data Synthesis and Statistical Analysis

Where a sufficient number of studies reported the same outcome using compatible measures, random-effects meta-analysis was performed using the DerSimonian–Laird estimator for between-study variance (τ^2^). Effect sizes were expressed as mean differences (MDs) with 95% confidence intervals (CIs) for continuous outcomes. For radiographic buccal bone plate thickness outcomes, pooled analyses were based on final follow-up values rather than calculated change-from-baseline scores because several included studies did not report the paired statistical parameters required for robust change-score meta-analysis. Where these parameters were missing, the corresponding authors were contacted, but the required data could not be obtained. Statistical heterogeneity was assessed using the I^2^ statistic and Cochran’s Q test. Heterogeneity was categorized as low (I^2^ 0–25%), moderate (26–50%), substantial (51–75%), or considerable (>75%). Where meta-analysis was not possible due to clinical or methodological heterogeneity, a narrative synthesis was conducted, grouping studies by comparator type and outcome domain. All meta-analyses were conducted in R version 4.3.2 using the meta package.

## 3. Results

### 3.1. Study Selection

**Figure 1:**
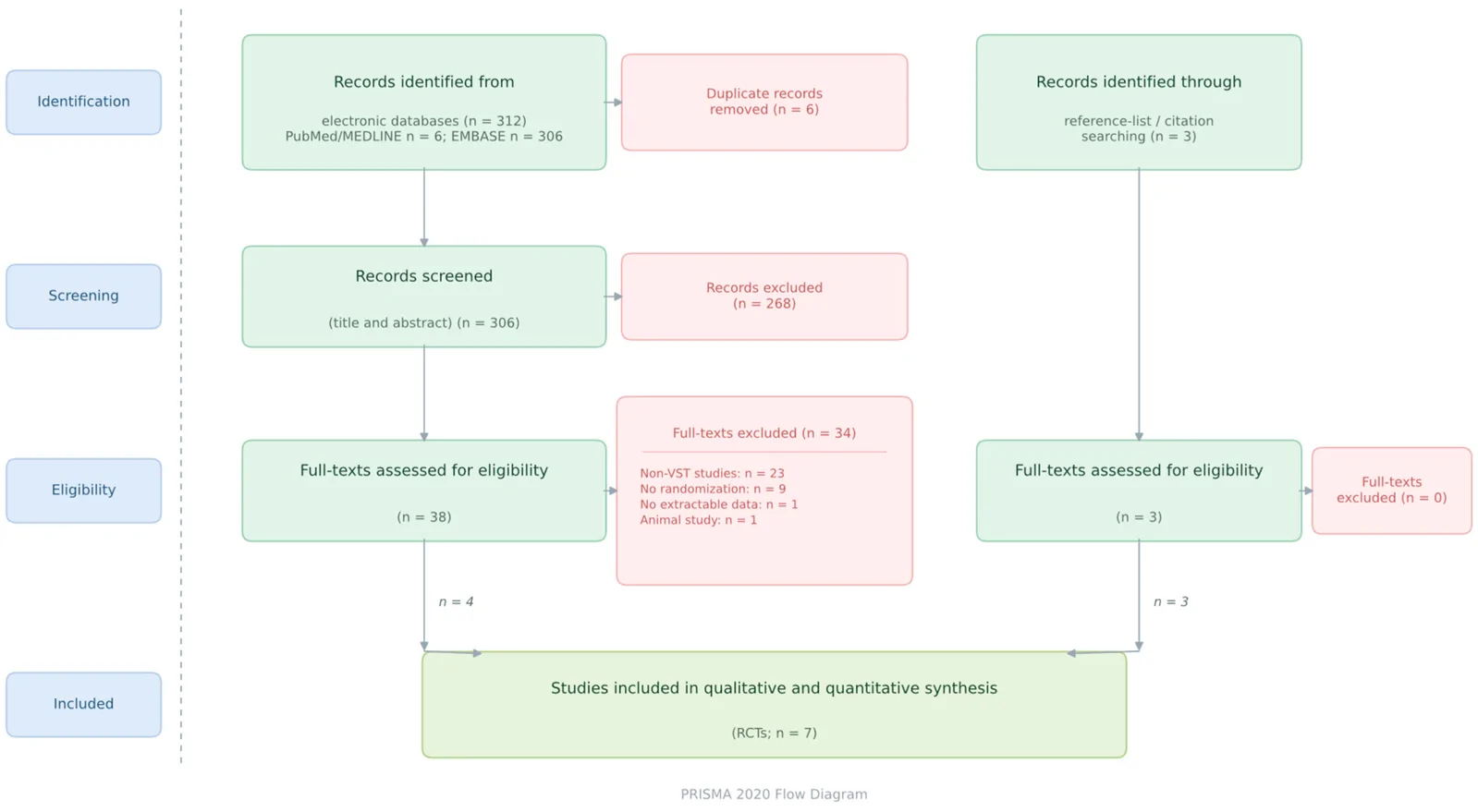
PRISMA 2020 flow diagram of study selection.

The PubMed/MEDLINE search returned 6 records, and the EMBASE search returned 306 records (last searched January 2026), giving 312 records in total. The six PubMed/MEDLINE records were also indexed in EMBASE and were removed as duplicates, leaving 306 unique records for screening. Following title and abstract screening, 268 records were excluded, and the full texts of 38 reports were assessed; of these, 34 were excluded (non-VST immediate implant studies, n = 23; cohort or case-series studies without randomization, n = 9; no extractable comparative data, n = 1; animal study, n = 1), and 4 RCTs met the inclusion criteria. The grey-literature search of ClinicalTrials.gov and ICTRP identified no additional eligible studies; however, reference-list (citation) searching of the included trials and relevant systematic reviews identified 3 further eligible RCTs. In total, 7 RCTs were included in the qualitative and quantitative synthesis. The study selection process is summarized in Figure 1.

### 3.2. Characteristics of Included Studies

The seven included RCTs were published between 2022 and 2024 and collectively enrolled 186 participants across 15 intervention arms, ranging from 20 to 40 participants per trial. All trials were conducted in Egypt, with procedures performed at private implant practice settings or university dental outpatient clinics in Alexandria, Tanta, and Cairo. This distribution across several centers does not, however, indicate investigator independence: the trials share overlapping senior authorship and originate from the group that developed and refined VST and are therefore best regarded as one closely related research network rather than independent replications. Participant ages ranged from 18 to 58 years across trials, with a predominance of female participants in most studies. Key characteristics of all included studies are summarized in Table 1.

All trials targeted the maxillary esthetic zone encompassing anterior teeth and premolars. Four trials enrolled patients with compromised Type II extraction sockets, characterized by partial or complete labial bone plate deficiency with intact overlying soft tissues. Two trials enrolled patients with intact but thin-walled Type I sockets, and one trial enrolled Type II sockets in the context of an extraction technique comparison. Common inclusion criteria across all studies comprised a minimum of 3 mm of sound apical bone for implant anchorage, a primary stability threshold of at least 30 Ncm, non-smoking status, and absence of uncontrolled systemic disease or acute periapical infection at the surgical site. Notably, although the review’s eligibility permitted both infected and non-infected sites, none of the included trials enrolled acutely infected sockets.

Although six of the seven included trials originated from overlapping investigator networks and clinical settings in Egypt, cross-examination of recruitment periods, sample sizes, socket classifications, and clinical questions confirmed that all trials enrolled independent, non-overlapping patient cohorts. No participants were shared across studies. The trials addressed seven distinct clinical questions within the VST framework, each with a separately defined population, eligibility criteria, and comparator arm, further supporting cohort independence.

These questions were as follows. One trial assessed VST against the contour-augmentation technique; the Buser protocol of early implant placement with simultaneous guided bone regeneration for compromised Type II extraction sockets. One trial assessed the effect of grafting the jumping gap with particulate bone versus leaving it to heal by blood clot formation alone. One trial assessed a novel vestibular root extraction technique against conventional incisal atraumatic extraction as the upstream extraction step performed immediately before VST. One trial assessed VST against partial extraction therapy (socket shield technique) for IIP in intact thin-walled Type I sockets. One trial assessed the VST bone-shielding concept against the dual-zone grafting technique in intact thin-walled Type I sockets. One trial assessed three grafting materials used within the VST protocol: a collagen plug soaked in blood, demineralized bone matrix (DBM) Grafton, and a particulate mixture of autogenous cortical chips combined with deproteinized bovine bone mineral (MinerOss X). One trial assessed acellular dermal matrix against autogenous connective tissue graft as soft tissue augmentation adjuncts, combined with VST and bone grafting, in Type II sockets with a thin gingival phenotype.

All trials employed platform-switched tapered implants with computer-aided design/computer-aided manufacturing (CAD/CAM)-guided placement, a flexible equine cortical membrane shield stabilized with membrane tacks, and customized PEEK healing abutments to preserve socket architecture during the healing phase. Final restorations were full-contour zirconia crowns with an S-shaped transmucosal emergence profile, delivered at 2–3 months post-implant placement in the majority of trials. Buccal bone plate thickness was measured by CBCT at baseline before implant placement and before any grafting procedure, as well as at final follow-up, in all trials. All trials reported their CBCT acquisition parameters (including voxel size, tube voltage, and field of view) and the imaging software used for linear measurement, and in every trial, the outcome was measured by an assessor who was blinded to group allocation and, where stated, calibrated. Pink Esthetic Score was assessed in all trials from standardized clinical photographs by two independent blinded examiners with reported inter-examiner agreement values of ≥0.82 kappa across all trials (Table 2).

### 3.3. Risk of Bias Assessment

**Figure 2:**
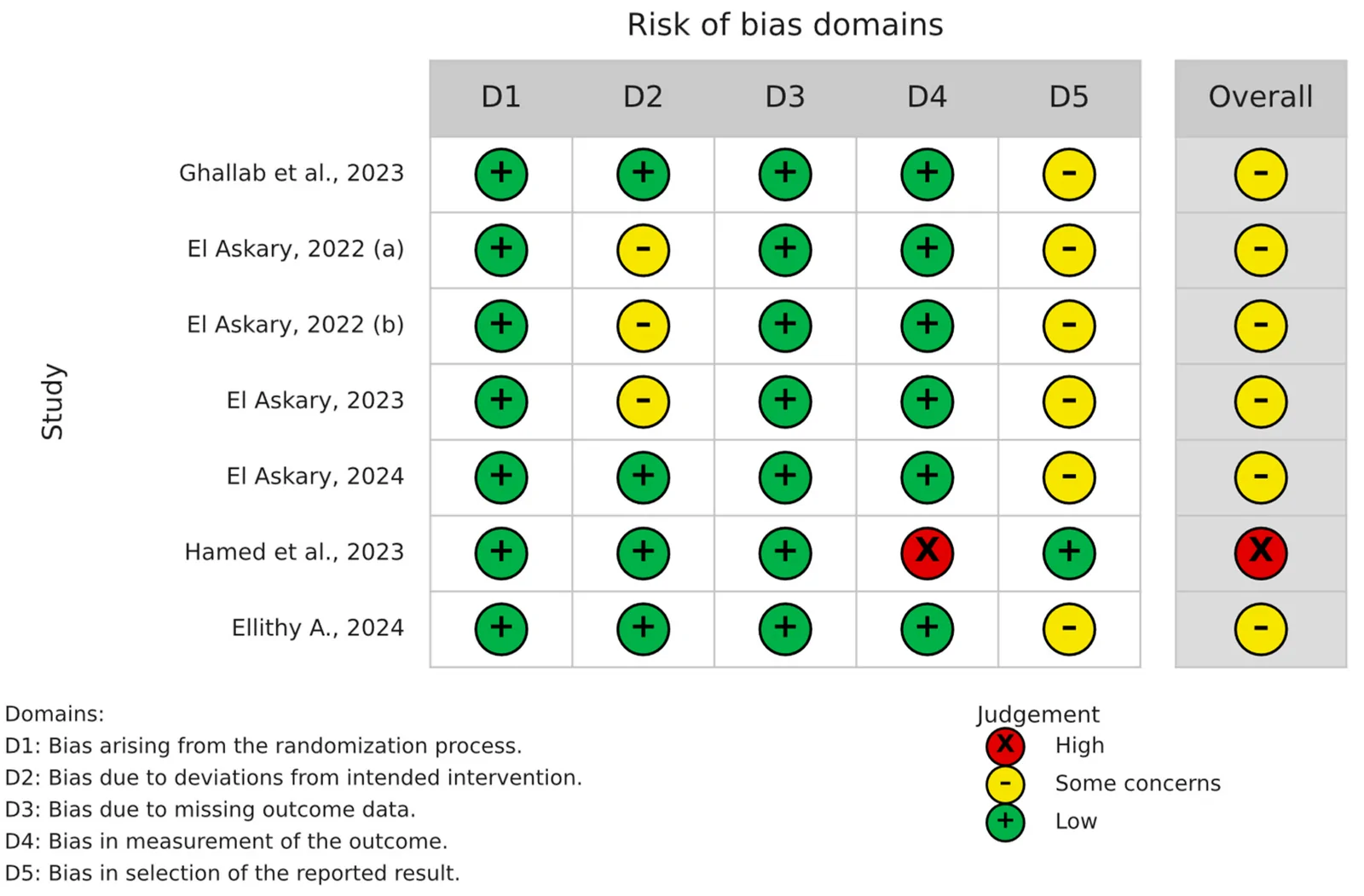
Traffic-light plot of the risk-of-bias assessment for individual randomized controlled trials using the Cochrane Risk of Bias 2 (RoB 2) tool [17,18,23,24,25,26,27].

**Figure 3:**
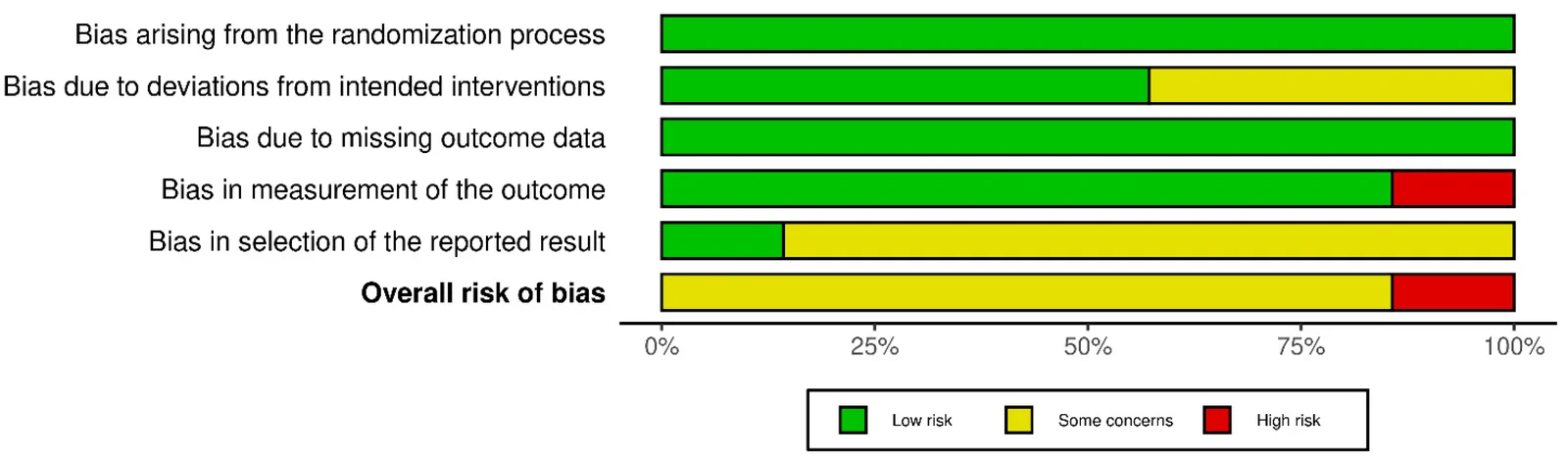
Risk of bias summary graph of the included randomized controlled trials assessed using the Cochrane Risk of Bias 2 (RoB 2) tool.

The risk of bias of the included randomized controlled trials was assessed using the Cochrane Risk of Bias 2 (RoB 2) tool across five domains: bias arising from the randomization process, bias due to deviations from intended interventions, bias due to missing outcome data, bias in measurement of the outcome, and bias in selection of the reported result. Of the seven included trials, six were judged as raising some concerns overall [18,23,24,25,26,27], while one trial was judged at high overall risk of bias Hamed et al., 2023 [17]. The randomization process was considered adequate across all included studies. Concerns in the deviations and selection domains arose primarily from the inability to blind operators because of the surgical nature of the interventions, exclusion of a randomized implant failure in one study, and the absence of publicly accessible pre-specified statistical analysis plans or protocols confirming outcome-specific analyses before unblinded outcome data were available. The high-risk judgment for Hamed et al., 2023 [17] was driven by concerns in the measurement domain, where the absence of blinded outcome assessment introduced potential detection bias, particularly for subjective esthetic and soft tissue outcomes. A graphical summary of the risk-of-bias assessment is presented in Figure 2 and Figure 3, and the complete domain-level assessment, including responses to all signaling questions and supporting justifications for each study, is provided in Supplementary Table S1.

### 3.4. Qualitative Synthesis: Outcome-by-Outcome Narrative

#### 3.4.1. Implant Survival

Implant survival was 100% across six of the seven included trials at their respective final follow-up endpoints. The single exception was Elaskary et al., 2022 [23], in which one implant failed in the VST arm, yielding a per-protocol survival rate of 95% (19/20) in that group and 100% in the contoaugmentation comparator arm. Across all seven trials, 185 of 186 were successfully osseointegrated and functional at final follow-up. No postoperative complications, including shield exposure, peri-implantitis, wound dehiscence, or membrane exposure, were reported in any trial. These data collectively suggest the clinical reliability and safety of the VST protocol across a range of socket types and adjunct materials.

#### 3.4.2. Primary Outcome: Buccal Bone Plate Thickness

Every VST-treated arm showed a statistically significant intra-group increase in buccal bone plate thickness from baseline to final follow-up, irrespective of comparator or grafting material. When VST was compared against an alternative surgical technique, however, between-group differences were generally not statistically significant. As these measurements are radiographic, they reflect an increase in radiographically detectable buccal bone dimension and, in the absence of histological confirmation, cannot be equated with new bone formation.

**Comparisons of VST with alternative surgical techniques.**

In the trial comparing VST with early implant placement and contour augmentation Elaskary et al. 2022 [23], both techniques produced significant intra-group gains at all three levels, with no significant between-group difference at 12 months: apical, 2.21 ± 0.93 mm (VST) versus 2.73 ± 1.35 mm (*p* = 0.261); mid-root, 2.10 ± 0.68 mm versus 2.47 ± 1.08 mm (*p* = 0.318); and crestal, 1.97 ± 0.82 mm versus 1.95 ± 0.77 mm (*p* = 0.715).

In the trial comparing VST with partial extraction therapy, Elaskary et al. 2023 [24], both groups showed significant intra-group gains at all three levels at 6 months, with no significant between-group difference at any level (all *p* ≥ 0.05). Final values in the VST group were 1.87 ± 0.99 mm crestally, 2.24 ± 0.91 mm at the mid-root, and 3.32 ± 1.72 mm apically; corresponding PET values were 1.95 ± 0.95 mm, 1.75 ± 1.00 mm, and 2.40 ± 0.50 mm.

In the trial comparing the VST bone-shielding approach with the dual-zone concept in thin-walled (Type I) sockets, Elaskary et al. 2024 [18], the bone-shielding group showed significantly greater final buccal bone thickness at the crestal (0.82 ± 0.40 mm versus 0.34 ± 0.33 mm) and mid-root (1.64 ± 0.50 mm versus 0.83 ± 0.52 mm) levels; the apical difference was smaller and not statistically significant (2.12 ± 0.56 mm versus 1.80 ± 0.33 mm). Buccal bone thickness changes are displayed in Table 2.

**Comparisons within the VST protocol.**

Three trials evaluated variations within VST rather than VST against an alternative technique and are reported here as internal protocol comparisons only. In the grafted versus ungrafted jumping-gap trial, Elaskary et al. 2022 [26], grafting the gap with particulate bone produced significantly greater buccal bone thickness than clot-only healing at the mid-root (2.95 ± 0.97 mm versus 1.82 ± 0.64 mm; *p* = 0.003) and apical (3.75 ± 1.30 mm versus 2.03 ± 0.81 mm; *p* = 0.002) levels, with no significant difference crestally (2.17 ± 1.03 mm versus 2.07 ± 0.81 mm; *p* = 0.847).

In the trial comparing three grafting materials, all applied within the VST protocol Hamed et al. 2023 [17], each material produced a significant intra-group increase at 12 months (collagen plug, *p* = 0.008; DBM/Grafton, *p* = 0.024; particulate autogenous + DBBM, *p* = 0.027), with median final values of 1.01, 1.11, and 1.44 mm, respectively, and no significant between-material difference at 6 or 12 months (*p* = 0.898 and *p* = 0.523).

Ghallab et al., 2023 [25], comparing a vestibular extraction approach with conventional incisal extraction, did not report buccal bone plate thickness as extractable mean ± SD values and therefore did not contribute to this outcome.

In the trial comparing acellular dermal matrix (ADM) with connective tissue graft (CTG), both within the VST protocol Ellithy et al., 2024. [27], Both modalities produced significant intra-group increases at all three levels (all *p* < 0.001) with no significant between-group difference in thickness; buccal bone height at 12 months was, however, significantly greater in the ADM group (13.86 ± 1.56 mm) than in the CTG group (12.14 ± 1.58 mm; *p* = 0.028).

#### 3.4.3. Secondary Outcome: Peri-Implant Soft Tissue Changes

The most pronounced inter-group differences in soft tissue outcomes were observed in Elaskary et al., 2022 [23], where VST produced significantly less mid-facial recession (−0.53 ± 1.17 mm) than early placement with contour augmentation (−1.87 ± 0.69 mm; *p* < 0.001), a mean difference of 1.34 mm favoring VST. Horizontal (bucco-lingual) soft tissue loss was likewise significantly lower with VST than with contour augmentation in the same trial (0.82 ± 0.95 mm versus 1.84 ± 0.88 mm; *p* = 0.001). In Elaskary et al., 2024 [18], the bone-shielding group showed minimal mid-facial recession (−0.15 ± 0.35 mm) versus greater recession in the dual-zone group (−0.44 ± 0.23 mm; *p* = 0.026). In Ghallab et al., 2023 [25], the vestibular root extraction group exhibited genuine soft tissue creeping at the mid-facial margin (+0.39 ± 0.64 mm), contrasting with recession in the conventional extraction group (−0.32 ± 0.68 mm; *p* = 0.006). In the trial comparing VST to PET Elaskary et al., 2023 [24], mid-facial changes were near-zero in the VST group (+0.01 ± 0.73 mm) and minor in the PET group (−0.20 ± 0.10 mm; *p* = 0.33).

Mesial and distal papilla changes were reported in three trials. Both papillae showed significantly less change with VST than contour augmentation (mesial: 0.64 vs. 1.20 mm, *p* = 0.023; distal: 0.56 vs. 1.26 mm, *p* = 0.001) in Elaskary et al., 2022 [23]. In Ghallab et al., 2023 [25], the vestibular root extraction group showed minimal papillary creeping at both the mesial (+0.26 mm) and distal (+0.05 mm) papillae, whereas the conventional extraction group showed recession at both sites (mesial: −0.39 mm; distal: −0.37 mm), with significant inter-group differences (all *p* < 0.05). In Elaskary et al., 2024 [18], papillary recession was smaller in the VST (bone-shielding) group than the dual-zone group at both the mesial (−0.12 vs. −0.23 mm) and distal (−0.19 vs. −0.59 mm) papillae.

Mucosal thickness was reported only in Ellithy et al. 2024 [27]. Both ADM and CTG produced statistically significant increases in coronal mucosal thickness from baseline (ADM: 1.26 → 3.40 ± 0.45 mm; CTG: 1.30 → 3.48 ± 0.54 mm; both *p* < 0.001), with no significant inter-group difference at any time point.

#### 3.4.4. Secondary Outcome: Pink Esthetic Score

PES was reported in four of seven trials. Across all trials and all groups, PES values ranged from 10.29 to 13.17 at final follow-up, consistently reflecting good to excellent peri-implant esthetic outcomes.

In Ghallab et al., 2023 [25], total PES was significantly higher in the vestibular root extraction group (12.67 ± 1.59) than in the conventional incisal extraction group (11.40 ± 1.40; *p* = 0.03). In Elaskary et al. 2023 [24], total PES was 12.67 ± 1.3 (VST) versus 13.17 ± 1.19 (PET), with no significant difference (*p* = 0.33). In Elaskary et al., 2024 [18], total PES strongly favored bone shielding (mean 12.05) over the dual-zone technique (mean 10.29). In Hamed et al. 2023 [17], the median PES values at 12 months were 12.00 (collagen), 11.00 (Grafton), and 13.00 (MinerOss X), with no statistically significant inter-group differences (*p* = 0.388); 91.6% of all cases achieved a PES > 10.

#### 3.4.5. Secondary Outcome: Peri-Implant Probing Depth

Peri-implant probing depth was formally reported only in Elaskary et al. 2023 [24], where mean values were 2.16 ± 0.44 mm (VST) and 2.08 ± 1.02 mm (PET) at 6 months (*p* = 0.79), both consistent with healthy peri-implant sulci.

### 3.5. Meta-Analysis

**Figure 4:**
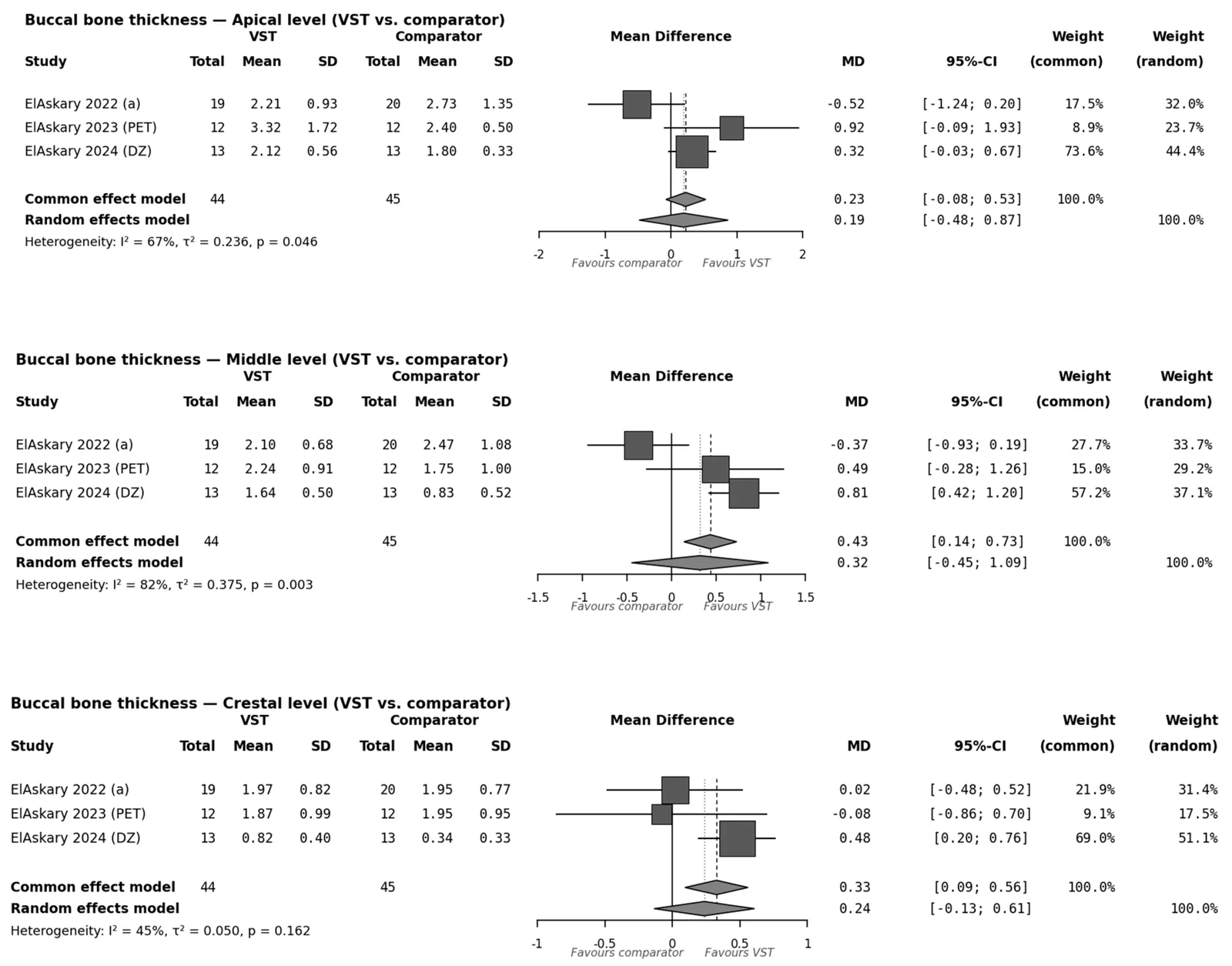
Forest plots of the meta-analysis comparing vestibular socket therapy (VST) with control protocols for buccal bone thickness [18,23,26].

Quantitative synthesis was performed for the primary outcome of final buccal bone plate thickness across three anatomical levels: crestal, mid-root, and apical. Pooling was restricted to the three trials in which VST was compared against a distinct alternative surgical technique: early implant placement with contour augmentation Elaskary et al. 2022 [23], partial extraction therapy Elaskary et al. 2023 [24], and the dual-zone concept Elaskary et al. 2024 [18]. Four trials compared variations within the VST protocol—the extraction technique Ghallab et al. 2023 [25], grafted versus ungrafted jumping gap Elaskary et al. 2022 [26], three grafting materials Hamed et al. 2023 [17], and acellular dermal matrix versus connective tissue graft Ellithy et al. 2024 [27]—and were not pooled, as both arms of each received VST and therefore did not represent a VST versus alternative contrast. Ghallab et al. 2023 [25] and Hamed et al. 2023 [17] additionally did not report buccal bone thickness as mean ± SD; the corresponding authors were contacted, but the required data could not be obtained. Pooled analyses used final follow-up CBCT values rather than change-from-baseline scores, as the paired parameters required for change-score meta-analysis were not consistently reported. A random-effects (DerSimonian–Laird) model was applied. Follow-up was 12 months for the contour-augmentation and dual-zone comparisons and 6 months for the PET comparison. Because only one trial was available per comparator type, subgroup meta-analysis and formal sensitivity analysis were not feasible. Pooled results are illustrated in Figure 4.

Crestal Bone Thickness. The pooled random-effects estimate for final crestal bone thickness was 0.24 mm (95% CI −0.13 to 0.61) in favor of VST, which was not statistically significant, as the confidence interval included the null value (Figure 4). Under the common-effect model, the same contrast was statistically significant (0.33 mm, 95% CI 0.09 to 0.56); given the moderate between-study heterogeneity, however, the random-effects estimate is the more appropriate summary, and even that sub-millimeter difference is of uncertain clinical importance. Heterogeneity was moderate (I^2^ = 45%).

Mid-Root Bone Thickness. The pooled estimate was +0.32 mm (95% CI −0.45 to 1.09), which did not reach statistical significance (Figure 4). Substantial heterogeneity was present (I^2^ = 82%), and this result should not be interpreted beyond a non-significant numerical trend.

*Apical Bone Thickness.* The pooled estimate was +0.19 mm (95% CI −0.48 to 0.87); again, not statistically significant (Figure 4). Heterogeneity was substantial (I^2^ = 67%), and this result should likewise be regarded as a non-significant numerical trend.

Overall Interpretation. Across all three anatomical levels, the meta-analysis showed no statistically significant difference in final buccal bone plate thickness between VST and its comparators; every pooled confidence interval crossed the null value. The point estimates consistently, though modestly, favored VST (+0.19 to +0.32 mm), but none reached significance, and heterogeneity was moderate to substantial (I^2^ = 45%, 82%, and 67% at the crestal, mid-root, and apical levels, respectively), consistent with the clinically distinct comparators pooled. Taken together, the meta-analytic evidence does not provide statistically reliable support for a buccal bone plate thickness advantage of VST over this heterogeneous comparator set at any level. These findings should be regarded as preliminary and hypothesis-generating and require confirmation in independent, adequately powered trials with homogeneous comparators.

#### Certainty of the Evidence (GRADE)

The certainty of evidence for the three pooled outcomes was assessed using the GRADE approach and is summarized in the Summary of Findings table (Table 3). As all outcomes were derived from randomized controlled trials, each began at high certainty. For all three outcomes, certainty was downgraded for risk of bias (−1), as most trials were judged to have “some concerns” and one was at high risk on RoB 2, compounded by the overlapping authorship between the review and the included trials; for imprecision (−1), as every pooled confidence interval crossed the null value and spanned clinically meaningful differences in both directions on a small total sample (n = 89), and for publication bias (−1, strongly suspected), as all pooled trials originated from a single closely related investigator network. For the mid-root and apical outcomes, an additional downgrade for inconsistency (−1) was applied given the substantial heterogeneity (I^2^ = 82% and 67%). Accordingly, certainty was rated very low for all three outcomes: crestal (+0.24 mm; 95% CI −0.13 to 0.61), mid-root (+0.32 mm; 95% CI −0.45 to 1.09), and apical (+0.19 mm; 95% CI −0.48 to 0.87).

## 4. Discussion

This systematic review and meta-analysis represents the first synthesis of randomized controlled trial evidence exclusively evaluating VST in conjunction with IIP. Seven RCTs enrolling 186 participants were included, comparing VST both against established alternative techniques and across internal protocol variations. VST was associated with high implant survival and favorable short-term soft tissue and esthetic outcomes, with buccal bone plate thickness comparable to that of established comparators across a range of socket types and clinical scenarios. These observations warrant cautious optimism, tempered by an evidence base limited in scope, geographic diversity, and operator independence.

IIP in the esthetic zone remains one of the most technically demanding procedures in implant dentistry, with post-extraction buccal bone resorption the principal biological obstacle to predictable long-term esthetic stability Couso-Queiruga et al., 2021 [7]. Horizontal bone loss of 3.5–5 mm and vertical reduction of 1.5–2 mm have been reported within the first six months of extraction, and IIP alone does not arrest this physiologic remodeling Fan et al., 2025 [28]. VST addresses this through a minimally invasive vestibular tunnel that reconstructs the deficient buccal plate with a flexible cortical membrane while preserving the crestal soft tissue envelope, which flap-based approaches inevitably disrupt.

The pooled implant survival of 99.5% is consistent with, and in some respects exceeds, rates reported in broader reviews of IIP in compromised and infected sockets Amid et al. 2023 [29]. The low rate of biological complications, including shield exposure, peri-implantitis, and wound dehiscence, is particularly noteworthy against the PET literature, where shield exposure and associated peri-implant complications have been reported in 2–19% of cases across larger retrospective series Gluckman et al. 2018 [30]; Gluckman & Salama 2023 [31].

The primary meta-analytic finding was that VST did not differ significantly from its comparators in final crestal buccal bone plate thickness. The pooled point estimates modestly favored VST at all three levels but did not reach statistical significance: at the crestal level, the effect was small and of uncertain direction. Confirmation in adequately powered, independent trials is required before it can support clinical decisions. This matters because crestal bone level governs the overlying mucosal architecture: crestal loss produces a predictable apical shift of the peri-implant gingival margin Breunig et al. 2024 [32]; da Silva et al. 2025 [33]. This link was directly demonstrated in ElAskary et al. 2022 [23], where 12-month coronal bone thickness was a significant independent predictor of mid-facial soft tissue change in a multiple linear regression model.

The significant differences in final crestal and mid-root bone thickness between the bone-shielding and dual-zone groups in Elaskary et al. 2024 [18] reinforce the interpretation that the cortical membrane barrier, rather than graft volume alone, is the critical determinant of crestal bone preservation.

The substantial heterogeneity at the mid-root (I2 = 82%) and apical (I2 = 67%) levels, together with the absence of a significant pooled effect, likely reflects biological and methodological variability across trials, in particular the clinically distinct comparators, variable socket morphologies, and grafting protocols. Even at the crestal level, where heterogeneity was more moderate (I2 = 45%), the pooled estimate did not reach significance, so no anatomical level provided statistically reliable evidence of a bone-thickness advantage for VST.

Although soft tissue outcomes could not be pooled owing to heterogeneity in measurement methods, they converged across all included trials in favor of VST. Replacing crestal incisions and periosteal reflection with a vestibular tunnel may prevent the apical shift of the keratinized mucosal band seen with flap-based approaches, a rationale supported by tunneling flaps in mucogingival surgery Aroca et al. 2013 [34]; Aroca et al. 2026 [35] and by evidence that minimizing periosteal disruption during IIP yields long-term mucosal stability Jain et al. 2024 [36].

PES values ranged from 10.29 to 13.17 across all arms and trials, comparable with the weighted mean PES of approximately 12.27 reported in a recent systematic review of the socket shield technique Wu et al. 2022 [37].

The pooled comparators, early implant placement with contour augmentation, the dual-zone concept, and PET, are surgically distinct but share a single clinical objective: rehabilitating compromised or thin-walled anterior extraction sockets while preserving peri-implant soft tissue architecture and esthetic outcomes. They were therefore considered clinically relevant comparators reflecting the actual decision space in the anterior maxilla.

Several limitations must be acknowledged. All seven trials were conducted in Egypt by a closely related investigator group, raising concerns about geographic and operator generalizability that only independent multicenter replication can address. In addition, the originator of the VST technique co-authored the present review and is the lead or senior author of most of the included trials; although study selection, data extraction, and risk-of-bias assessment were performed independently by reviewers who took no part in the primary trials, this overlap is an inherent limitation, and independent replication by unrelated groups is required before the findings can be considered confirmed. With one trial per comparator type, subgroup meta-analysis was precluded, so the pooled estimates reflect the average effect of VST against a heterogeneous comparator set, one that differs not only in surgical approach but also in biological mechanism, rather than a head-to-head effect against any single protocol. The crestal estimate itself was non-significant, and even its 0.24 mm point value would require clinical contextualization given the precision limitations of CBCT-based measurement. Because several trials did not report the paired parameters required for a change-score synthesis, final follow-up buccal bone plate thickness was pooled rather than change from baseline; as baseline thickness varied between individuals and arms, residual baseline imbalance cannot be excluded as an influence on the pooled estimates.

Inconsistent reporting of baseline determinants (keratinized mucosa width, gingival phenotype, jumping-gap dimension) precluded assessment of when VST is most and least beneficial. Individual trials were small, ranging from 20 to 40 participants, limiting power at the mid-root and apical levels, and too few studies were available for funnel-plot assessment of publication bias. The search was also restricted to two bibliographic databases (PubMed/MEDLINE and EMBASE); although a grey-literature search of ClinicalTrials.gov and ICTRP returned no additional eligible studies, the omission of other sources such as Scopus, Web of Science, and the Cochrane CENTRAL register may have missed eligible reports. Follow-up reached only 12 months, leaving longer-term stability unconfirmed at the RCT level. Using the GRADE approach, certainty was rated very low for all three bone-thickness outcomes, downgraded for high risk of bias and strongly suspected publication bias arising from the concentration of trials within a single investigator network; the learning curve associated with VST may further limit reproducibility in less experienced settings. Finally, the search was limited to two databases and no formal sensitivity analyses were conducted, limitations to be addressed as the evidence base expands.

While the present evidence does not yet support formal clinical recommendations, several preliminary observations merit attention as hypotheses for future investigation. A single RCT suggested that grafting the jumping gap with particulate bone may enhance final buccal bone thickness at the mid-root and apical levels compared with blood clot alone, which, if replicated, could inform protocol standardization. A single RCT found VST equivalent to PET for short-term esthetic and bone outcomes in intact thin-walled Type I sockets, suggesting a biologically plausible alternative for this indication, though its applicability to broader indications requires dedicated evidence. A single RCT found ADM equivalent to CTG for mucosal thickening while producing greater facial bone height at 12 months, which, if confirmed, could avoid palatal donor-site morbidity. Each derives from a single small trial within one investigator network and cannot alone modify practice; independent, adequately powered, multicenter trials are required before any can inform clinical recommendations.

## 5. Conclusions

Within the limitations of this systematic review and meta-analysis, VST demonstrated favorable short-term clinical and esthetic outcomes when used in conjunction with immediate implant placement across included trials. Implant survival was high, VST did not differ significantly from comparator techniques in final buccal bone plate thickness at any level, and the pooled estimates showed moderate to substantial heterogeneity. The certainty of evidence across all three bone-thickness outcomes was very low. All included trials originated from a single investigator network, and follow-up durations were limited to 12 months, with insufficient reporting of baseline anatomical variables, which restricts the generalizability of the findings. The internal consistency observed within this single research environment should not be equated with independent external validation. Accordingly, VST should be regarded as a promising and biologically plausible technique. Independent, adequately powered, multicenter randomized controlled trials with longer follow-up and standardized outcome reporting are needed before firm clinical recommendations can be made.

## Figures and Tables

**Table 1:** Characteristics of included studies.

Study	Socket Type	Intervention (Test Arm)	Comparator (Control Arm)	*n* (Per arm)	Follow-Up
**VST versus an alternative surgical technique (pooled in the meta-analysis)**					
**ElAskary 2022a [23]**	Type II (deficient)	VST + immediate implant	Early placement + contour augmentation (GBR)	20/20	12 mo
**ElAskary 2023 [24]** **(PET)**	Type I (intact, thin)	VST + immediate implant	Partial extraction therapy	12/12	6 mo
**ElAskary 2024 [18]** **(dual-zone)**	Type I (intact, thin)	VST bone shielding + immediate implant	Dual-zone grafting + immediate implant	13/13	12 mo
**Comparisons within the VST protocol (narrative synthesis only)**					
**Ghallab 2023 [25]**	Type II (deficient)	VST + vestibular root extraction	VST + conventional incisal extraction	15/15	12 mo
**ElAskary 2022b [26]**	Type I/II	VST + grafted jumping gap	VST + ungrafted jumping gap (blood clot)	11/11	12 mo
**Hamed 2023 [17]**	Type II	VST + collagen plug (Grp I)	VST + DBM/Grafton (II); VST + autogenous chips + DBBM (III)	8/8/8	12 mo
**Ellithy 2024 [27]**	Type II	VST + ADM	VST + CTG	10/10	12 mo

All trials were conducted in the maxillary esthetic zone in non-infected sockets with immediate implant placement using an equine cortical membrane (OsteoBiol Lamina, Tecnoss) fixed with tacks (0.6 mm in all trials, except 1.0 mm in ElAskary 2024 [18]) and CBCT-based bone assessment. ElAskary 2022a [23] randomized 40 participants (20 per arm); one implant in the VST arm failed and was excluded from the per-protocol bone-thickness analysis (analyzed: n = 19 test/20 control). In Ghallab 2023 [25], both arms received VST; the trial compared the extraction technique, not VST against an alternative. VST, vestibular socket therapy; GBR, guided bone regeneration; PET, partial extraction therapy; DBM, demineralized bone matrix; DBBM, deproteinized bovine bone mineral; ADM, acellular dermal matrix; CTG, connective tissue graft.

**Table 2:** Buccal plate of bone thickness changes.

Study (Year)	Group	Level	Baseline Thickness, Mean ± SD (mm)	Final Thickness, Mean ± SD (mm): Forest-Plot Input ★	Measured Structure/Method †
**ElAskary (2022a) [23]**	Test (VST)	Crestal	NR	1.97 ± 0.82	Buccal plate of bone thickness by: CBCT †
	Control (early)	Crestal	NR	1.95 ± 0.77	
	Test (VST)	Middle	0.62 ± 0.67	2.10 ± 0.68	
	Control (early)	Middle	1.12 ± 1.26	2.47 ± 1.08	
	Test (VST)	Apical	1.53 ± 1.29	2.21 ± 0.93	
	Control (early)	Apical	1.97 ± 1.69	2.73 ± 1.35	
**ElAskary (2023) [24]**	Test (VST)	Crestal	0.53 ± 0.60	1.87 ± 0.99	Buccal plate of bone thickness by: CBCT †
	Control (PET)	Crestal	0.42 ± 0.36	1.95 ± 0.95	
	Test (VST)	Middle	0.62 ± 0.54	2.24 ± 0.91	
	Control (PET)	Middle	0.48 ± 0.54	1.75 ± 1.00	
	Test (VST)	Apical	1.64 ± 1.27	3.32 ± 1.72	
	Control (PET)	Apical	1.82 ± 0.87	2.40 ± 0.50	
**ElAskary (2024) [18]**	Test (VST)	Crestal	0.79 ± 0.39	0.82 ± 0.40	Buccal plate of bone thickness by: CBCT
	Control (dual zone)	Crestal	0.73 ± 0.26	0.34 ± 0.33	
	Test (VST)	Middle	1.06 ± 0.44	1.64 ± 0.50	
	Control (dual zone)	Middle	1.01 ± 0.31	0.83 ± 0.52	
	Test (VST)	Apical	1.26 ± 0.61	2.12 ± 0.56	
	Contro (dual zone)l	Apical	1.16 ± 0.23	1.80 ± 0.33	
**ElAskary (2022b) [26]**	Test (VST+ grafted jumping gap)	Crestal	0.54 ± 0.59	2.17 ± 1.03	Buccal plate of bone thickness by: CBCT
	Control (non grafted jumping gap)	Crestal	0.59 ± 0.49	2.07 ± 0.81	
	Test (VST + grafted jumping gap)	Middle	0.91 ± 0.97	2.95 ± 0.97	
	Control (non grafted jumping gap)	Middle	0.37 ± 0.41	1.82 ± 0.64	
	Test (VST + grafted jumping gap)	Apical	2.91 ± 1.89	3.75 ± 1.30	
	Control (non grafted jumping gap)	Apical	1.40 ± 1.17	2.03 ± 0.81	
**Ellithy A (2024) [27]**	Test (VST + ADM)	Crestal	0.31 ± 0.16	2.19 ± 0.72	Buccal plate of bone thickness by; CBCT †
	Control (VST + CTG)	Crestal	0.22 ± 0.08	1.72 ± 0.89	
	Test (VST + ADM)	Middle	0.63 ± 0.14	2.30 ± 0.85	
	Contr ol (VST + CTG)	Middle	0.63 ± 0.32	2.29 ± 0.63	
	Test (VST + ADM)	Apical	0.45 ± 0.21	2.04 ± 1.41	
	Control (VST + CTG)	Apical	0.64 ± 0.21	2.56 ± 0.68	

**Table 3:** Summary of findings: certainty of evidence using the GRADE approach.

№ of Studies	Study Design	Certainty Assessment					№ of Patients		Effect		Certainty	Importance
		Risk of Bias	Inconsistency	Indirectness	Imprecision	Other Considerations	Intervention	Comparison	Relative (95% CI)	Absolute (95% CI)		
**Crestal bone thickness (follow-up: 6–12 months; assessed with: CBCT, mm)**												
3	Randomised trials	Serious ^a^	Not serious	Not serious	Serious ^c^	Publication bias strongly suspected ^d^	44	45	—	MD 0.24 mm higher (0.13 lower to 0.61 higher)	⨁◯◯◯Very low	CRITICAL
**Mid-root bone thickness (follow-up: 6–12 months; assessed with: CBCT, mm)**												
3	Randomised trials	Serious ^a^	Serious ^b^	Not serious	Serious ^c^	Publication bias strongly suspected ^d^	44	45	—	MD 0.32 mm higher (0.45 lower to 1.09 higher)	⨁◯◯◯Very low	CRITICAL
**Apical bone thickness (follow-up: 6–12 months; assessed with: CBCT, mm)**												
3	Randomised trials	Serious ^a^	Serious ^b^	Not serious	Serious ^c^	Publication bias strongly suspected ^d^	44	45	—	MD 0.19 mm higher (0.48 lower to 0.87 higher)	⨁◯◯◯Very low	CRITICAL

The three pooled trials each contribute all three levels: ElAskary 2022a [23] (analyzed: n = 19/20; 12 mo), ElAskary 2023 [24] vs. PET (12/12; 6 mo), and ElAskary 2024 [18] vs. dual-zone (13/13; 12 mo); total 44 VST vs. 45 comparators. Effect estimates are from random-effects (DerSimonian–Laird) meta-analysis of final follow-up values. ^a.^ Risk of bias: most trials had “some concerns” on RoB 2 (one at high risk), and the reviewing and originating authorship overlap. ^b.^ Inconsistency: substantial statistical heterogeneity (I^2^ = 82% mid-root; 67% apical) with effect estimates differing in direction across trials. ^c.^ Imprecision: the 95% CI includes no effect and spans clinically important differences in both directions on a small total sample (n = 89). ^d.^ Publication bias strongly suspected: all pooled trials originate from a single closely related investigator network, including the technique’s originator. ^e.^ The pooled comparators (contour augmentation, PET, dual zone) are clinically distinct techniques and follow-up differed (6 vs. 12 months), representing clinical heterogeneity in the comparison.

## Data Availability

The datasets generated and analyzed during the current study are available from the corresponding author on reasonable request.

## Supplementary Materials

The following supporting information can be downloaded at: https://www.mdpi.com/article/10.3390/dj14090538/s1. File S1: PubMed/MEDLINE electronic search strategy, including all search terms, Boolean operators, field tags, filters, and date parameters; File S2: EMBASE electronic search strategy, including all search terms, Boolean operators, field tags, filters, and date parameters; Table S1: Domain-level risk-of-bias assessment (Cochrane RoB 2), including responses to all signaling questions and supporting justifications for each included study; Table S2: PRISMA 2020 checklist.
