# Supplementary material for: Clinical and Esthetic Outcomes of Vestibular Socket Therapy: A Systematic Review and Meta-Analysis
Source: Dent J (Basel). 2026 Aug 29;14(9):538. doi: 10.3390/dj14090538 (PMC13605699; doi:10.3390/dj14090538)
Supplement: Supplementary file 1 [file dentistry-14-00538-s001.zip › Supplementary file S1-PuBMED.pdf]

("Vestibular Socket Therapy"[Title/Abstract] OR "VST"[Title/Abstract])
AND
("Dental Implantation"[MeSH Terms] OR "Dental Implants"[MeSH Terms] OR "immediate implant"[Title/Abstract] OR "immediate implant placement"[Title/Abstract] OR "immediate implantation"[Title/Abstract])
AND
("Alveolar Bone Loss"[MeSH Terms] OR "Alveolar Ridge Augmentation"[MeSH Terms] OR "buccal bone"[Title/Abstract] OR "labial bone"[Title/Abstract] OR "facial bone"[Title/Abstract] OR "bone thickness"[Title/Abstract] OR "bone regeneration"[Title/Abstract])
AND
("Gingival Recession"[MeSH Terms] OR "soft tissue"[Title/Abstract] OR "gingival recession"[Title/Abstract] OR "papilla"[Title/Abstract] OR "esthetic outcome"[Title/Abstract] OR "esthetic score"[Title/Abstract] OR "Pink Esthetic Score"[Title/Abstract] OR "PES"[Title/Abstract])

No language restrictions applied
Database: PubMed/MEDLINE
Search date: January 2026
ClinicalTrials.gov Search terms: "vestibular socket therapy"; "VST implant" Date searched: January 2026 Results reviewed: titles and brief summaries of registered trials
