# Supplementary material for: Clinical and Esthetic Outcomes of Vestibular Socket Therapy: A Systematic Review and Meta-Analysis
Source: Dent J (Basel). 2026 Aug 29;14(9):538. doi: 10.3390/dj14090538 (PMC13605699; doi:10.3390/dj14090538)
Supplement: Supplementary file 1 [file dentistry-14-00538-s001.zip › Supplementary file S2-EMBASE.pdf]

('vestibular socket therapy':ab,ti OR 'VST':ab,ti)
AND
('dental implant'/exp OR 'immediate dental implant placement':ab,ti OR 'immediate implant':ab,ti OR 'immediate implant placement':ab,ti)
AND
('alveolar bone'/exp OR 'buccal bone':ab,ti OR 'labial bone':ab,ti OR 'facial bone thickness':ab,ti OR 'bone regeneration'/exp OR 'guided bone regeneration':ab,ti)
AND
('gingival recession'/exp OR 'soft tissue':ab,ti OR 'papilla':ab,ti OR 'esthetic outcome':ab,ti OR 'pink esthetic score':ab,ti OR 'PES':ab,ti)

Filters: Publication date: 2020–2026
No language restrictions applied
Database: EMBASE (via Elsevier)
Search date: January 2026
