# Supplementary material for: Clinical and Esthetic Outcomes of Vestibular Socket Therapy: A Systematic Review and Meta-Analysis
Source: Dent J (Basel). 2026 Aug 29;14(9):538. doi: 10.3390/dj14090538 (PMC13605699; doi:10.3390/dj14090538)
Supplement: Supplementary file 1 [file dentistry-14-00538-s001.zip › supplementary Table S1.pdf]

Ghallab et al., 2023

Record ID 5 | Reviewer M.I | Comparison: VST extraction vs. periotome extraction | Outcome: Vertical soft tissue changes and Pink Esthetic Score (PES)| Overall judgement: **Some concerns**

RoB 2 domain	Judgement	Supporting justification
**D1. Randomisation process**	**Low**	The allocation sequence was random; the allocation sequence was concealed until participants were enrolled and assigned to interventions. Baseline differences did not suggest a problem with the randomisation process.
**D2. Deviations from intended interventions**	**Low**	Due to the nature of the surgical interventions, neither participants nor clinical investigators could be blinded. There is no indication in the trial report of any deviations from the intended interventions caused by the trial context. The study analysed outcomes using appropriate statistical methods consistent with the effect of assignment to intervention (intention-to-treat principles): the authors used independent-sample t-tests, paired t-tests, Mann–Whitney, and Wilcoxon tests depending on data distribution. All participants were included; no other exclusions or missing data issues were reported. The statistical plan was clearly described, and analyses compared groups as assigned.
**D3. Missing outcome data**	**Low**	Data for the outcomes were available for all randomised participants
**D4. Measurement of the outcome**	**Low**	There is no indication that outcome measurement could have differed between the intervention groups. Outcome assessors were not aware of which intervention participants received.
**D5. Selection of the reported result**	**Low**	The article does not report whether analyses were conducted according to a pre-specified statistical analysis plan finalized before unblinded outcome data were available. Although the reported outcomes and analyses appear consistent, no publicly accessible protocol or statistical analysis plan confirming pre-specification of the outcome analyses was identified. Therefore, some concerns were assigned for bias in selection of the reported result.
**Overall risk of bias**	**Some concerns**	Domains 1–4 were judged low risk. Domain 5 raised some concerns because no publicly accessible pre-specified statistical analysis plan confirming outcome-specific analyses before unblinded outcome data were available was identified. In addition, the assessed outcomes included subjective esthetic and soft tissue measurements. Therefore, the overall judgement was some concerns.

El Askary, 2022b

Record ID 7 | Reviewer YS | Comparison: VST + jumping gap vs. VST | Outcome: Bone changes | Overall judgement: **Some concerns**

RoB 2 domain	Judgement	Supporting justification
**D1. Randomisation process**	Low	The allocation sequence was random; the allocation sequence was concealed until participants were enrolled and assigned to interventions. Baseline differences did not suggest a problem with the randomisation process.
**D2. Deviations from intended interventions**	Some concerns	Participants were aware of the intervention they received, and the people delivering the interventions (the clinicians/surgeons) were aware of the participants' assigned intervention. In the article, there is no evidence that knowledge of assignment or other features of the trial context led to deviations from the intended interventions: all 22 participants were randomised (11 per group) and all completed the assigned intervention; the CONSORT flow shows no crossovers, no discontinuations, and no protocol violations. Although the investigator (surgeon) was not blinded, the participants were blinded and the outcome assessors were blinded, and the report does not describe any changes in care or delivery that would constitute deviations arising from the trial context. The article used statistical methods that are appropriate for estimating the effect of assignment to intervention in a randomised controlled trial.
**D3. Missing outcome data**	Low	Outcome data were available for all randomised participants.
**D4. Measurement of the outcome**	Low	The article used appropriate, validated, and standardised methods to measure the primary outcome (labial bone thickness), with no indication of inappropriateness. There is no indication in the article that measurement or ascertainment of the outcome could have differed between the intervention groups. The outcome assessors were not aware of the intervention received by study participants.
**D5. Selection of the reported result**	Some concerns	The article does not report whether the data were analysed according to a pre-specified analysis plan (SAP) finalised before outcome data were unblinded. There is no indication in the article that multiple eligible outcome measurements existed within the same outcome domain from which the authors could have selectively chosen results. The article reports a single, consistent analysis pathway and does not indicate that the authors had multiple eligible analytic approaches from which they could have selectively chosen results. The statistical plan lists specific tests used for each purpose and applies them uniformly: independent t-test (age), chi-square (sex), Mann–Whitney U for between-group comparisons of bone thickness, and Wilcoxon signed-rank for within-group pre/post comparisons; normality was checked with Shapiro–Wilk. There is no mention of alternative adjusted models, alternative covariance structures, per-protocol vs. ITT contrasts, or multiple alternative time-point/model specifications that could enable selective reporting.
**Overall risk of bias**	Some concerns	Domains 1, 3, and 4 were judged low risk. Domain 2 raised some concerns because operators and participants could not be blinded given the surgical nature of the intervention, although no deviations arising from the trial context were reported and an appropriate intention-to-treat–consistent analysis was used. Domain 5 raised some concerns because no pre-specified analysis plan was reported, although a single consistent analysis pathway was applied across outcomes. The overall judgement is therefore some concerns.

El Askary, 2022a

Record ID 8 | Reviewer YS | Comparison: VST vs. early implant placement + contour augmentation | Outcome: Bone changes | Overall judgement: Some concerns

RoB 2 domain	Judgement	Supporting justification
**D1. Randomisation process**	Low	The allocation sequence was generated randomly using an online randomisation tool (Random.org), as stated in the study's Randomisation and blinding section. This indicates that participants were assigned to groups based on a truly random, computer-generated sequence, fulfilling criteria for low risk of bias in sequence generation. The study reports that age, gender distribution, and tooth location were not statistically different between the two groups (all $p > 0.05$), indicating that the groups were comparable at baseline and that randomisation worked as intended.
**D2. Deviations from intended interventions**	Some concerns	Due to the surgical nature of the interventions, operators and participants were not blinded. One implant failure in the test group was excluded from the final analysis, resulting in a per-protocol exclusion rather than a strict intention-to-treat analysis. Although no additional protocol deviations or crossovers were reported, exclusion of a randomized participant with a clinically relevant failure introduces some concerns regarding bias arising from deviations from intended interventions..
**D3. Missing outcome data**	Low	Data for this outcome were available for nearly all randomised participants.
**D4. Measurement of the outcome**	Low	There is no indication that outcome measurement could have differed between the intervention groups. Outcome assessors were not aware of which intervention participants received.
**D5. Selection of the reported result**	Some concerns	The article does not report whether analyses were conducted according to a pre-specified statistical analysis plan finalized before unblinded outcome data were available. Although the reported analyses appeared internally consistent, no publicly accessible protocol or statistical analysis plan confirming pre-specification of outcome analyses was identified. Therefore, some concerns were assigned for bias in selection of the reported result.
**Overall risk of bias**	Some concerns	Domains 1, 3, and 4 were judged low risk. Domain 2 raised some concerns because one implant failure in the intervention group was excluded from the final analysis, resulting in a per-protocol exclusion. Domain 5 also raised some concerns because no publicly accessible pre-specified statistical analysis plan was identified. Therefore, the overall judgement was some concerns..

El Askary, 2023

Record ID 9 | Reviewer YS | Comparison: VST vs. partial extraction therapy (PET) | Outcome: Bone changes | Overall judgement: **Some concerns**

RoB 2 domain	Judgement	Supporting justification
**D1. Randomisation process**	Low	The allocation sequence was random. The allocation sequence was concealed until participants were enrolled and assigned to interventions. Baseline differences did not suggest any issue with the randomisation process.
**D2. Deviations from intended interventions**	Some concerns	Participants were aware of their assigned intervention, and carers and people delivering the interventions were aware of participants' assigned intervention. There is no evidence that knowledge of assignment or other trial-context factors caused deviations from the intended interventions. The CONSORT flow shows: 24 randomised (12 per group) → 24 received allocated interventions → 24 analysed, with no crossovers, discontinuations, or protocol violations reported. The article used appropriate statistical methods for estimating the effect of assignment to intervention in a two-arm randomised controlled trial. The authors reported unpaired Student's t-tests for comparing continuous outcomes between groups (e.g., PES, bone thickness, soft-tissue changes), chi-square tests for categorical variables (e.g., gender distribution), and descriptive statistics including means, SDs, mean differences, and 95% confidence intervals.
**D3. Missing outcome data**	Low	Outcome data were available for all randomised participants.
**D4. Measurement of the outcome**	Low	There is no indication in the article that the method used to measure outcomes was inappropriate; the study employed validated, standardised, and widely accepted measurement techniques for all primary and secondary outcomes. There is no indication in the article that measurement or ascertainment of outcomes differed between the VST and partial extraction therapy groups. Outcome assessors were not aware of which intervention each participant received.
**D5. Selection of the reported result**	Some concerns	The article does not report whether the analysis was performed according to a pre-specified statistical analysis plan (SAP) finalised before unblinded outcome data were available. There is no evidence in the article that the authors had multiple eligible outcome measurements within the same outcome domain from which they could have selectively reported favourable results. There is no indication in the article that the authors conducted or had access to multiple eligible analyses of the data from which they could have selectively chosen favourable results.
**Overall risk of bias**	Some concerns	Domains 1, 3, 4, and 5 were judged low risk. Domain 2 raised some concerns because operators and participants could not be blinded given the surgical nature of the intervention, although no deviations arising from the trial context were reported and an appropriate intention-to-treat–consistent analysis was used. The overall judgement is therefore some concerns.

El Askary, 2024

Record ID 10 | Reviewer YS | Comparison: VST vs. dual-zone technique | Outcome: Bone change | Overall judgement: **Some concerns**

RoB 2 domain	Judgement	Supporting justification
**D1. Randomisation process**	Low	The allocation sequence was random. The allocation sequence was concealed until participants were enrolled and assigned. Baseline characteristics between the two groups did not show statistically significant differences, indicating no evidence of a problem with the randomisation process.
**D2. Deviations from intended interventions**	Low	Participants were aware of the intervention they received, and carers and people delivering the interventions were aware of participants' assigned intervention. Even though participants and surgeons were not blinded, the article provides no indication of any deviations from intended interventions that occurred because of the trial context. Appropriate statistical methods were used to estimate the effect of assignment to intervention: unpaired Student's t-test for continuous outcomes and chi-square test for categorical variables, with reporting of means, SDs, mean differences, and 95% confidence intervals.
**D3. Missing outcome data**	Low	Data for the outcomes were available for all randomised participants.
**D4. Measurement of the outcome**	Low	The article used appropriate, validated, and standardised methods for measuring all primary and secondary outcomes. There is no indication that the measurement methods were inappropriate. There is no indication that measurement or ascertainment of outcomes differed between the two intervention groups in the article. Outcome assessors in the article were not aware of participants' assigned interventions.
**D5. Selection of the reported result**	Some concerns	The article does not report whether the analyses were conducted according to a pre-specified statistical analysis plan finalized before unblinded outcome data were available. Although the study reported a single, internally consistent analysis pathway and no evidence of selective outcome reporting was identified, no publicly accessible protocol or statistical analysis plan confirming pre-specification of outcome analyses was available. Therefore, some concerns were assigned for bias in selection of the reported result..
**Overall risk of bias**	Some concerns	Domains 1–4 were judged low risk. Domain 5 raised some concerns because no publicly accessible pre-specified statistical analysis plan confirming outcome-specific analyses before unblinded outcome data were available was identified. Therefore, the overall judgement was some concerns.

Hamed et al., 2023

Record ID 11 | Reviewer M.I | Comparison: Different grafting techniques vs. CTG | Outcome: Bone changes | Overall judgement: **High**

RoB 2 domain	Judgement	Supporting justification
**D1. Randomisation process**	Low	The allocation sequence was random. The allocation sequence was concealed until participants were enrolled and assigned to interventions. Baseline differences did not suggest any issue with the randomisation process.
**D2. Deviations from intended interventions**	Low	he surgery is unblinded by nature — a single investigator performed all surgical and restorative procedures, and participants/providers knew the graft assigned. However, for the *effect of assignment*, what matters is whether deviations arose from the trial context and whether analysis was ITT-consistent. The CONSORT flow shows 24 randomised (8/group), all receiving the allocated intervention, no crossovers or discontinuations, and all 24 analysed as allocated. No trial-context deviations are reported.
**D3. Missing outcome data**	Low	Data for the outcomes were available for all randomised participants
**D4. Measurement of the outcome**	High	The primary outcome (Pink Esthetic Score) is a subjective, observer-rated esthetic index. The paper describes no blinding of outcome assessors for any outcome, names no independent assessor, and states all procedures were done by a single investigator — so outcome assessment was very likely performed with knowledge of the assigned graft. For a subjective primary outcome, that awareness can plausibly influence scoring, and the same exposure affects the assessor-dependent secondary measures (PML by digital superimposition; FBT on CBCT)
**D5. Selection of the reported result**	Low	The trial was prospectively registered (NCT04957654, 12 July 2021), the authors state the full protocol is available, and PES/PML/FBT are defined. A single consistent analysis pathway is used (normality test → Kruskal–Wallis between groups; Friedman/Wilcoxon within groups), applied uniformly with no alternative models presented
**Overall risk of bias**	High	At least one domain (D4) is High, so under the RoB 2 algorithm the **overall is High**

Ellithy A, 2024

Record ID 13 | Reviewer YS | Comparison: VST vs. VST (ADM vs. CTG) | Outcome: Bone changes | Overall judgement: **Some concerns**

RoB 2 domain	Judgement	Supporting justification
**D1. Randomisation process**	Low	A single investigator performed a two-column sequence generation using an online tool (www.random.org); the allocation sequence was concealed until participants were enrolled and assigned to their intervention groups. Baseline characteristics between the ADM and CTG groups did not show any statistically significant differences, indicating that randomisation successfully created comparable groups and that there is no evidence of a problem with the randomisation process.

RoB 2 domain	Judgement	Supporting justification
**D2. Deviations from intended interventions**	Low	Participants were aware of their assigned intervention during the trial, and carers and people delivering the interventions were aware of participants' assigned intervention. Although both participants and care providers (implantologist) were not blinded, the article reports no deviations from the intended interventions that arose because of the trial context. Normality was checked with the Shapiro–Wilk test before selecting tests. Because most continuous variables were non-normally distributed, between-group comparisons used Mann–Whitney U tests (ADM vs. CTG) rather than parametric t-tests. Within-group (longitudinal) comparisons of repeated measures across baseline, 6 months, and 12 months were analysed with Friedman's test, followed — when significant — by Dunn–Šidák pair-wise comparisons, with Bonferroni adjustment applied to control for multiple testing. Baseline categorical characteristics (e.g., sex, tooth type, reasons for extraction) were compared with chi-square tests.
**D3. Missing outcome data**	Low	Data for this outcome were available for all randomised participants.
**D4. Measurement of the outcome**	Low	All outcome-measurement methods used in the article are standard, validated, and appropriate for assessing soft- and hard-tissue changes in implant dentistry. There is no indication that the measurement methods were inappropriate. There is no indication that measurement or ascertainment of outcomes differed between the two intervention groups (ADM vs. CTG). The study used identical, standardised, and blinded measurement procedures for both groups. Outcome assessors were not aware of participants' assigned interventions; the paper states that all outcomes were measured by an experienced calibrated radiologist who was blinded to the patients' data and allocation.
**D5. Selection of the reported result**	Some concerns	The article does not state whether the statistical analysis followed a pre-specified analysis plan (SAP) finalised before unblinded outcome data became available. There is no indication in the article that multiple eligible outcome measurements existed within the same outcome domain from which the authors could have selectively reported results. The study used one clearly defined measurement method per outcome domain, with no alternative scales, definitions, or time points that could introduce selective reporting bias. The article reports a single, consistent analysis pathway aligned with its small sample and mostly non-normal data.
**Overall risk of bias**	Some concerns	Domains 1–4 were judged low risk. Domain 5 raised some concerns because no pre-specified analysis plan was reported, although a single consistent analysis pathway was applied and outcome assessment was blinded. The overall judgement is therefore some concerns.
