# Supplementary material for: Clinical and Esthetic Outcomes of Vestibular Socket Therapy: A Systematic Review and Meta-Analysis
Source: Dent J (Basel). 2026 Aug 29;14(9):538. doi: 10.3390/dj14090538 (PMC13605699; doi:10.3390/dj14090538)
Supplement: Supplementary file 1 [file dentistry-14-00538-s001.zip › Supplementary Table S2-PRISMA checklist.pdf]

PRISMA 2020 Checklist

Manuscript: “Clinical and Aesthetic Outcomes of Vestibular Socket Therapy: A Systematic Review and Meta-Analysis”

This systematic review and meta-analysis was conducted and reported in accordance with the PRISMA 2020 statement. The table below maps each PRISMA 2020 checklist item to the section of the manuscript (or Supplementary Materials) in which it is reported. The review was prospectively registered in PROSPERO (CRD420261288724).

Section and Topic	Item #	Checklist item	Location where item is reported
**TITLE**			
**Title**	**1**	Identify the report as a systematic review.	Title (identified as “A Systematic Review and Meta-Analysis").
**ABSTRACT**			
**Abstract**	**2**	See the PRISMA 2020 for Abstracts checklist.	Abstract (structured: Introduction, Materials and Methods, Results, Conclusion).
**INTRODUCTION**			
**Rationale**	**3**	Describe the rationale for the review in the context of existing knowledge.	Introduction, paragraphs 1–5.
**Objectives**	**4**	Provide an explicit statement of the objective(s) or question(s) the review addresses.	Introduction, final paragraph; Methods – “Study Question and PICO Framework” (PICO question stated).
**METHODS**			
**Eligibility criteria**	**5**	Specify the inclusion and exclusion criteria for the review and how studies were grouped for the syntheses.	Methods – “Eligibility Criteria”; “Study Question and PICO Framework”; grouping by comparator type described in “Data Synthesis and Statistical Analysis.”
**Information sources**	**6**	Specify all databases, registers, websites, organisations, reference lists and other sources searched or consulted to identify studies. Specify the date when each source was last searched or consulted.	Methods – “Search Methods for the Identification of Studies” (PubMed/MEDLINE and EMBASE from inception to January 2026; ClinicalTrials.gov and WHO ICTRP; hand-searching of reference lists).
**Search strategy**	**7**	Present the full search strategies for all databases, registers and websites, including any filters and limits used.	Methods – “Search Methods for the Identification of Studies”; full strategies in Supplementary File 1.
**Selection process**	**8**	Specify the methods used to decide whether a study met the inclusion criteria of the review, including how many reviewers screened each record and each report retrieved, whether they worked independently, and if applicable, details of automation tools used in the process.	Methods – “Study Selection” (two independent reviewers, M.I. and Y.S.; disagreements resolved by consensus/third-reviewer arbitration; no automation tools used).
**Data collection process**	**9**	Specify the methods used to collect data from reports, including how many reviewers collected data from each report, whether they worked independently, any processes for obtaining or confirming data from study investigators, and if applicable, details of automation tools used in the process.	Methods – “Data Extraction” (two independent reviewers; pre-piloted standardized form; corresponding authors contacted for missing/unclear data; no automation tools used).
**Data items**	**10a**	List and define all outcomes for which data were sought. Specify whether all results that were compatible with each outcome domain in each study were sought (e.g. for all measures, time points, analyses), and if not, the methods used to decide which results to collect.	Methods – “Outcome Measures” (primary: final buccal bone plate thickness at three levels; secondary: implant survival, soft tissue stability, mid-facial recession/papilla position, PES, probing depth).

Section and Topic	Item #	Checklist item	Location where item is reported
	**10b**	List and define all other variables for which data were sought (e.g. participant and intervention characteristics, funding sources). Describe any assumptions made about any missing or unclear information.	Methods – "Data Extraction" (study identification, design/registration, setting, sample size, participant characteristics, comparator, implant/graft/membrane specifications, follow-up).
**Study risk of bias assessment**	**11**	Specify the methods used to assess risk of bias in the included studies, including details of the tool(s) used, how many reviewers assessed each study and whether they worked independently, and if applicable, details of automation tools used in the process.	Methods – "Risk of Bias and Quality Assessment" (Cochrane RoB 2; two independent reviewers, M.I. and Y.S.).
**Effect measures**	**12**	Specify for each outcome the effect measure(s) (e.g. risk ratio, mean difference) used in the synthesis or presentation of results.	Methods – "Data Synthesis and Statistical Analysis" (mean difference [MD] with 95% CI for continuous outcomes).
**Synthesis methods**	**13a**	Describe the processes used to decide which studies were eligible for each synthesis (e.g. tabulating the study intervention characteristics and comparing against the planned groups for each synthesis (item #5)).	Methods – "Data Synthesis and Statistical Analysis" (minimum of three studies with compatible measures; grouping by comparator type and outcome domain); Results – "Meta-Analysis."
	**13b**	Describe any methods required to prepare the data for presentation or synthesis, such as handling of missing summary statistics, or data conversions.	Methods – "Data Synthesis and Statistical Analysis"; Methods – "Outcome Measures" (pooling of final follow-up values rather than change-from-baseline; extraction of facial bone plate component only from ridge-width endpoints).
	**13c**	Describe any methods used to tabulate or visually display results of individual studies and syntheses.	Methods – "Data Synthesis and Statistical Analysis"; results displayed in Table 1, Table 2, and forest plots (Figure 4A–C).
	**13d**	Describe any methods used to synthesize results and provide a rationale for the choice(s). If meta-analysis was performed, describe the model(s), method(s) to identify the presence and extent of statistical heterogeneity, and software package(s) used.	Methods – "Data Synthesis and Statistical Analysis" (random-effects model, DerSimonian–Laird estimator; I^2 statistic and Cochran's Q; R v4.3.2, meta package).
	**13e**	Describe any methods used to explore possible causes of heterogeneity among study results (e.g. subgroup analysis, meta-regression).	Methods – "Data Synthesis and Statistical Analysis"; Results – "Meta-Analysis" (subgroup analysis by comparator type planned but not feasible – only one trial per comparator type; narrative synthesis used where pooling not possible).
	**13f**	Describe any sensitivity analyses conducted to assess robustness of the synthesized results.	Results – "Meta-Analysis" (no formal sensitivity analyses conducted, with rationale given – biological heterogeneity and single trial per comparator); Discussion (limitation acknowledged).
**Reporting bias assessment**	**14**	Describe any methods used to assess risk of bias due to missing results in a synthesis (arising from reporting biases).	Methods – "Risk of Bias and Quality Assessment" (publication bias considered within GRADE "other considerations" domain); Results – "Certainty of the Evidence (GRADE)"; funnel-plot assessment precluded by small study number (stated in Discussion).

Section and Topic	Item #	Checklist item	Location where item is reported
**Certainty assessment**	**15**	Describe any methods used to assess certainty (or confidence) in the body of evidence for an outcome.	Methods – "Risk of Bias and Quality Assessment" (GRADE approach across five domains; GRADEpro GDT).
**RESULTS**			
**Study selection**	**16a**	Describe the results of the search and selection process, from the number of records identified in the search to the number of studies included in the review, ideally using a flow diagram.	Results – "Study Selection" (312 records after de-duplication; 38 full-text; 7 RCTs included); Figure 1 (PRISMA 2020 flow diagram).
	**16b**	Cite studies that might appear to meet the inclusion criteria, but which were excluded, and explain why they were excluded.	Results – "Study Selection" (reasons for exclusion with counts: non-VST immediate implant studies n=23; non-randomized case series/cohort n=6; no extractable comparative data n=1; in vivo study n=1); Figure 1.
**Study characteristics**	**17**	Cite each included study and present its characteristics.	Results – "Characteristics of Included Studies"; Table 1.
**Risk of bias in studies**	**18**	Present assessments of risk of bias for each included study.	Results – "Risk of Bias Assessment"; Figures 2 and 3 (RoB 2 traffic-light and summary plots); Supplementary Table S1 (domain-level assessment).
**Results of individual studies**	**19**	For all outcomes, present, for each study: (a) summary statistics for each group (where appropriate) and (b) an effect estimate and its precision (e.g. confidence/credible interval), ideally using structured tables or plots.	Results – "Qualitative Synthesis: Outcome-by-Outcome Narrative" (means ± SD and p-values per group per study); Table 2; forest plots (Figure 4A–C).
**Results of syntheses**	**20a**	For each synthesis, briefly summarise the characteristics and risk of bias among contributing studies.	Results – "Meta-Analysis" (five trials contributing to pooled analyses identified; exclusions from pooling explained); "Risk of Bias Assessment."
	**20b**	Present results of all statistical syntheses conducted. If meta-analysis was done, present for each the summary estimate and its precision (e.g. confidence/credible interval) and measures of statistical heterogeneity. If comparing groups, describe the direction of the effect.	Results – "Meta-Analysis" (crestal MD +0.27 mm, 95% CI 0.00–0.54, I^2 3.5%; mid-root MD +0.41 mm, 95% CI −0.12–0.95, I^2 76.1%; apical MD +0.37 mm, 95% CI −0.44–1.18, I^2 78.7%); Figure 4A–C.
	**20c**	Present results of all investigations of possible causes of heterogeneity among study results.	Results – "Meta-Analysis" (heterogeneity at mid-root and apical levels attributed to comparator/protocol variability); Discussion (biological and methodological sources of heterogeneity). Subgroup analysis not feasible (one trial per comparator).
	**20d**	Present results of all sensitivity analyses conducted to assess the robustness of the synthesized results.	Results – "Meta-Analysis" (no formal sensitivity analyses conducted; rationale provided); Discussion (limitation acknowledged).
**Reporting biases**	**21**	Present assessments of risk of bias due to missing results (arising from reporting biases) for each synthesis assessed.	Results – "Certainty of the Evidence (GRADE)" (publication bias strongly suspected – all trials from a single investigator network; downgraded within GRADE); Discussion (funnel-plot analysis precluded by small number of studies).

Section and Topic	Item #	Checklist item	Location where item is reported
**Certainty of evidence**	**22**	Present assessments of certainty (or confidence) in the body of evidence for each outcome assessed.	Results – "Certainty of the Evidence (GRADE)" (low certainty for crestal; very low for mid-root and apical); Table 3 (Summary of Findings).
**DISCUSSION**			
**Discussion**	**23a**	Provide a general interpretation of the results in the context of other evidence.	Discussion, paragraphs 1–10 (findings interpreted against IIP, PET, socket-shield and contour-augmentation literature).
	**23b**	Discuss any limitations of the evidence included in the review.	Discussion – limitations paragraphs (single investigator network; heterogeneous comparators; modest sample sizes; 12-month maximum follow-up; incomplete baseline reporting).
	**23c**	Discuss any limitations of the review processes used.	Discussion – limitations paragraphs (only two databases; subgroup analysis and funnel-plot assessment not feasible; no formal sensitivity analyses).
	**23d**	Discuss implications of the results for practice, policy, and future research.	Discussion, final paragraphs; Conclusions (need for independent, adequately powered, multicentre RCTs with longer follow-up and standardized outcome reporting).
**OTHER INFORMATION**			
**Registration and protocol**	**24a**	Provide registration information for the review, including register name and registration number, or state that the review was not registered.	Title page (PROSPERO Registration No. CRD420261288724); Methods – "Protocol and Registration."
	**24b**	Indicate where the review protocol can be accessed, or state that a protocol was not prepared.	Methods – "Protocol and Registration" (protocol prospectively registered in PROSPERO, CRD420261288724, 19 January 2026; accessible via the PROSPERO register).
	**24c**	Describe and explain any amendments to information provided at registration or in the protocol.	Methods – "Protocol and Registration" (no amendments to the registered protocol were made).
**Support**	**25**	Describe sources of financial or non-financial support for the review, and the role of the funders or sponsors in the review.	Funding statement (no financial support received).
**Competing interests**	**26**	Declare any competing interests of review authors.	Conflict of Interest statement (authors declare no competing interests).
**Availability of data, code and other materials**	**27**	Report which of the following are publicly available and where they can be found: template data collection forms; data extracted from included studies; data used for all analyses; analytic code; any other materials used in the review.	Data Availability Statement (datasets available from the corresponding author on reasonable request); extracted data presented in Tables 1–2; search strategies in Supplementary File 1; RoB 2 domain-level assessments in Supplementary Table S1.

From: Page MJ, McKenzie JE, Bossuyt PM, Boutron I, Hoffmann TC, Mulrow CD, et al. The PRISMA 2020 statement: an updated guideline for reporting systematic reviews. BMJ 2021;372:n71. doi: 10.1136/bmj.n71.

This work is licensed under CC BY 4.0. To view a copy of this license, visit https://creativecommons.org/licenses/by/4.0/
